# Maternal immunization status and SARS-CoV-2 antibody transfer to neonates at birth

**DOI:** 10.3389/fped.2026.1824042

**Published:** 2026-07-01

**Authors:** J. Gómez-Carballo, R. González-Losa, L. Conde-Ferráez, C. Cen-Baas, N. Kantun-Moreno, H. Puerta-Guardo, J. A. Cruz-Cárdenas, M. E. G. Brunck, Y. Leal-Herrera, G. Valencia-Pacheco, M. García-Knight, G. Ayora-Talavera

**Affiliations:** 1 Centro de Investigaciones Regionales Dr. Hideyo Noguchi, Universidad Autónoma de Yucatán, Mérida, México; 2 Tecnológico de Monterrey, Escuela de Ingeniería y Ciencias, Monterrey, México; 3 Departamento de Inmunología, Instituto de Investigaciones Biomédicas, Universidad Nacional Autónoma de México (UNAM), México, México; 4 Centro Institucional de Capacitación y Registro de Cáncer (CICyRC), Unidad Médica de Alta Especialidad (UMAE) IMSS-Mérida, Yucatán, México

**Keywords:** cord blood antibodies, hybrid immunity, maternal antibodies, neonatal immune response, SARS-CoV-2 vaccination

## Abstract

**Introduction:**

Following the COVID-19 pandemic, hybrid immunity, arising from a combination of SARS-CoV-2 infection and vaccination, is widespread across populations, yet the dynamics of maternal antibody transfer to neonates in this context are not fully characterized.

**Methods:**

We conducted a cohort study of 117 pregnant women, assessing IgG antibodies against SARS-CoV-2 nucleoprotein and receptor-binding domain (RBD) in maternal, umbilical cord, and neonatal blood at delivery.

**Results:**

Using ELISA and pseudo virus neutralization assays, we found that 95% of mothers had detectable IgG, with 87% of neonates acquiring antibodies trans placentally. Hybrid immunity, detected in 65.5% of mothers, was associated with higher anti-RBD IgG levels in newborns compared to natural or vaccination-induced immunity alone. Neutralizing antibodies correlated positively with IgG titers, with umbilical cord samples showing the highest neutralization capacity.

**Discussion:**

These findings suggest that combined natural infection and vaccination in mothers enhance passive immunity in neonates, potentially improving early-life protection against SARS-CoV-2.

## 1 Introduction

The emergence of the Wuhan-Hu-1 variant of the SARS-CoV-2 virus in 2019 (1), was the cause of the COVID-19 pandemic. Six years after, SARS-CoV-2 continues to pose a public health concern. The emergence of viral variants has caused outbreaks of infection with variability in induced immunity (2, 3). Most individuals currently have what has been called “hybrid immunity” with adaptative immune responses, including antibodies elicited following natural infection and vaccination (4).

Pregnant women and their newborns are considered particularly vulnerable populations to SARS-CoV-2 infections. Studies have indicated that pregnant women face a higher risk of morbidity and mortality from COVID-19, which can lead to increased adverse pregnancy and birth outcomes. Although vertical transmission of SARS-CoV-2 appears to be rare or even non-existent, more sensitive and focused studies are needed to determine the presence of viral RNA in newborns (5). It is well established that maternal infection with various microorganisms can promote the transfer of antibodies to the fetus via the placenta or to the newborn through breast milk, thereby shaping the fetal and neonatal immune response, as has been demonstrated for diseases such as pertussis and influenza (6).

Six years following the start of the COVID-19 pandemic, the importance of vaccination during pregnancy is well established (7). Maternal vaccination provides greater persistence of antibodies in infants compared to immunity through natural infection. Additionally, vaccination has shown an increase in the rate of passive transfer of antibodies through the placenta and breast milk (8). However, in Mexico there is little information regarding the antibody response by natural immunity, by vaccination or hybrid in pregnant women and their newborn children. Thus, the objective of this work was to determine the serological levels of IgG antibodies transmitted to the newborn and their role in the neutralization of Wuhan-Hu-1 after three years of vaccination.

## 2 Materials and methods

### 2.1 Ethics statement and approval of the study

This study was approved by the Research Ethics Committee of the Dr. Agustín O'Horan General Hospital (Registration number: CEI-019-4-23) All participants provided verbal understanding and completed written informed consent.

### 2.2 Sample collection

This was a cross-sectional, observational and analytical study. Samples were collected from August 2023 to November 2024. Venous whole blood was collected from the mother-child pair and from the umbilical cord (UC) of the newborn (NB). The samples were collected during the stay in shared accommodation and in the case of the newborn, venous blood was taken 8 h after birth. Umbilical cord blood was collected during delivery or cesarean section. Samples from the mother and the NB were collected by venipuncture by authorized medical personnel with a volume of ≈5 mL in the case of the mothers and ≈500 µL for the NB. All samples were taken to the Virology laboratory of CIR-Biomedicas and processed to obtain serum by centrifugation at 3,500 rpm for 5 min. Serum was aliquoted and stored at −80 °C until use. Before use, serum was heat-inactivated in a biosafety cabinet at 56 °C for one hour. Positive and negative control sera from patients previously characterized with the presence and absence of antibodies against SARS-CoV-2, respectively, were used.

### 2.3 Generation of SARS-CoV-2 N and RBD proteins

Production of SARS-CoV-2 N and RBD proteins was according to the methodology proposed by Stadlbauer D. et al. 2020 (9) with some modifications. Briefly, HEK 293F cells were suspended at a concentration of 60  ×  106 cells in 20 mL (3 × 106 cells/mL) of Expi293 expression medium, with cell viability above 90%. For the transfection mixture, 1.2 mL of Opti-MEM was added to two sterile 15 mL polypropylene conical tubes: one tube received 20 μg (final concentration of 1 μg/μl in the total culture volume) of the respective plasmid DNA (for N or RBD), the other tube received 64 μl of ExpiFectamine transfection reagent. Subsequently, the content of both 15 mL tubes was mixed and incubated at room temperature for 10 min, after the incubation time, the transfection mixture was slowly added to the suspended cells while shaking in a circular manner and subsequently, the cells were incubated at 37 °C, 8% CO2 and constant shaking at 125 rpm. At 3 days post-transfection, cells were harvested and centrifuged for 10 min at 200× g, 4 °C to collect supernatant which was subsequently filtered through a 0.22 μm Stericup filter. Aliquots were made for freezing at −70 °C until use.

### 2.4 Determination of IgG antibodies (IgG Ab)

The ELISA test for IgG antibodies specific against the N and RBD proteins of the SARS-CoV-2 virus was performed according to the protocol standardized by Puerta-Guardo et al. in 2022 (10). Briefly, 96-well high-adhesion polystyrene plates (Immunolon® 4 HBX) were coated with approximately 37 nM of SARS-CoV-2 antigen (N or RBD) in 50 µL of sterile phosphate-buffered saline (PBS 1X, pH 7.4, Gibco), and incubated overnight at 4 °C. the next day, plates were washed in PBS and blocked for 1 h at 37 °C using a 100 mL of blocking buffer (2% low-fat milk, 0.05% Tween-20 in PBS). After blocking, three washes were given with PBS-0.05% Tween buffer. Then, 50 mL of twofold serially diluted human serum (starting from a 1:20 dilution) in buffer (1% non-fat milk in PBS-0.05% Tween), was added to each well and incubated at room temperature (RT) for 30 min with gentle shaking. After 3 washes to remove the excess serum dilution, the incubation was carried out with a secondary monoclonal antibody of mouse Anti-human IgG labeled with HRP (horseradish peroxidase) at a dilution of 1:10,000 in PBS-T 0.02% for 30 min at RT. For enzymatic detection, TMB (Sigma, Aldrich) was used as HRP substrate, and 50 µL 1N HCl solution was used to stop the enzymatic reaction. The absorbance reading was performed at 450 nm with a plate reader (Victor X3, 2030 multilabel reader, PerkinElmer). A pool of serums previously identified by a commercial kit was used as a positive serum control at a dilution of 1:1,000. As a negative serum control, a pool of sera from the virology laboratory serum bank collected prior to the pandemic and previously identified as negative by a commercial kit at a dilution of 1:1,000 was used. A cut-off point was calculated to discriminate between positive and negative sera based on the average of multiple measurements of the absorbance of the negative control sera plus or minus two times the standard deviation value. This cut-off point was performed for both measurements against N and RBD of SARS-CoV-2, resulting in values of ≥0.21 and ≥0.24 respectively. Hybrid immunity was defined as the detection of an IgG response to both N and RBD in the same sample/individual and a reported COVID-19 vaccination in the mother. Vaccine-derived immunity was defined by detection of IgG response to RBD but a lack of a response to N and a reported COVID-19 vaccination in the mother. Natural immunity was defined as the detection of an IgG response to both N and RBD and no reported COVID-19 vaccinations in the mother.

### 2.5 Neutralization assays with SARS-CoV-2 S lentiVP pseudoviruses

Each serum sample was analyzed with pseudoviruses expressing the S protein of the Wuhan-Hu-1 strain, using a 30% neutralization cutoff as established by Cruz-Cárdenas et al., 2022 (11). Three dilutions of 1:20, 1:160, and 1:1,280 were prepared in DMEM culture medium with 10% FBS from each serum sample. 100 µL of each dilution was incubated in duplicate with 15 pg of pseudovirus in 96-well plates for 1 h at 37 °C and 5% CO2. After incubation, 25,000 Vero cells were added to each well, and the plates were incubated for 24 h at 37 °C and 5% CO2. As a positive control for transduction, 15 pg of serum-free pseudovirus were incubated in the same number of Vero cells. Vero cells alone were used to evaluate the background luminescence. After 24 h, Nluc levels were measured according to the specifications of the Nano-Glo® Luciferase Assay System (PROMEGA) by the cell lysis method. Neutralization is described as the % inhibition of transduction, calculated as:

$$\begin{array}{rl} & \text{\%}\;\mathrm{Neut}\\ & =100\text{-}\frac{(\mathrm{average}\;\mathrm{RLU}\;\mathrm{of}\;\mathrm{sample}\;\text{-}\;\mathrm{average}\;\mathrm{RLU}\;\;\mathrm{of}\;\mathrm{target}\;\mathrm{cells})\times 100}{\;(\mathrm{average}\;\mathrm{RLU}\;\mathrm{of}\;\mathrm{infection}\;\text{-}\;\mathrm{average}\;\mathrm{RLU}\;\mathrm{of}\;\mathrm{target}\;\mathrm{cells})} \end{array}$$

### 2.6 Statistical analysis

The Wilcoxon test was used to compare the mean levels of Anti-N IgG vs. Anti-RBD IgG in each of the three sample groups (mother, UC, and NB). The Kruskal–Wallis ANOVA test was used to compare the geometric means of Anti-N IgG levels and Anti-RBD IgG levels among the three groups (mothers, CU, and NB). Assuming homogeneity of the samples and considering different sample sizes (n) for each group, a Welch ANOVA test was performed to determine the existence of significant differences between the geometric means of the EIA Index IgG Anti-RBD levels, with multiple comparisons using the Holm-Sidak method among the three groups classified according to their immunization status (natural immunity, vaccination-induced, or hybrid). To determine whether there were significant differences between the geometric means of the EIA Index IgG Anti-S-RBD levels in mothers, comparing the three groups segmented by vaccine dose with the control group of unvaccinated mothers, an ANOVA test was performed using Welch's method. This statistical design was also applied to the UC and NB samples, and to determine which comparison groups showed a significant difference, a Dunnett's multiple comparison test was run. All tests were performed using the statistical software GraphPad Prism 8.0.2 with a 95% confidence interval and a significance level of *p* < 0.05.

## 3 Results

**Figure 1:**
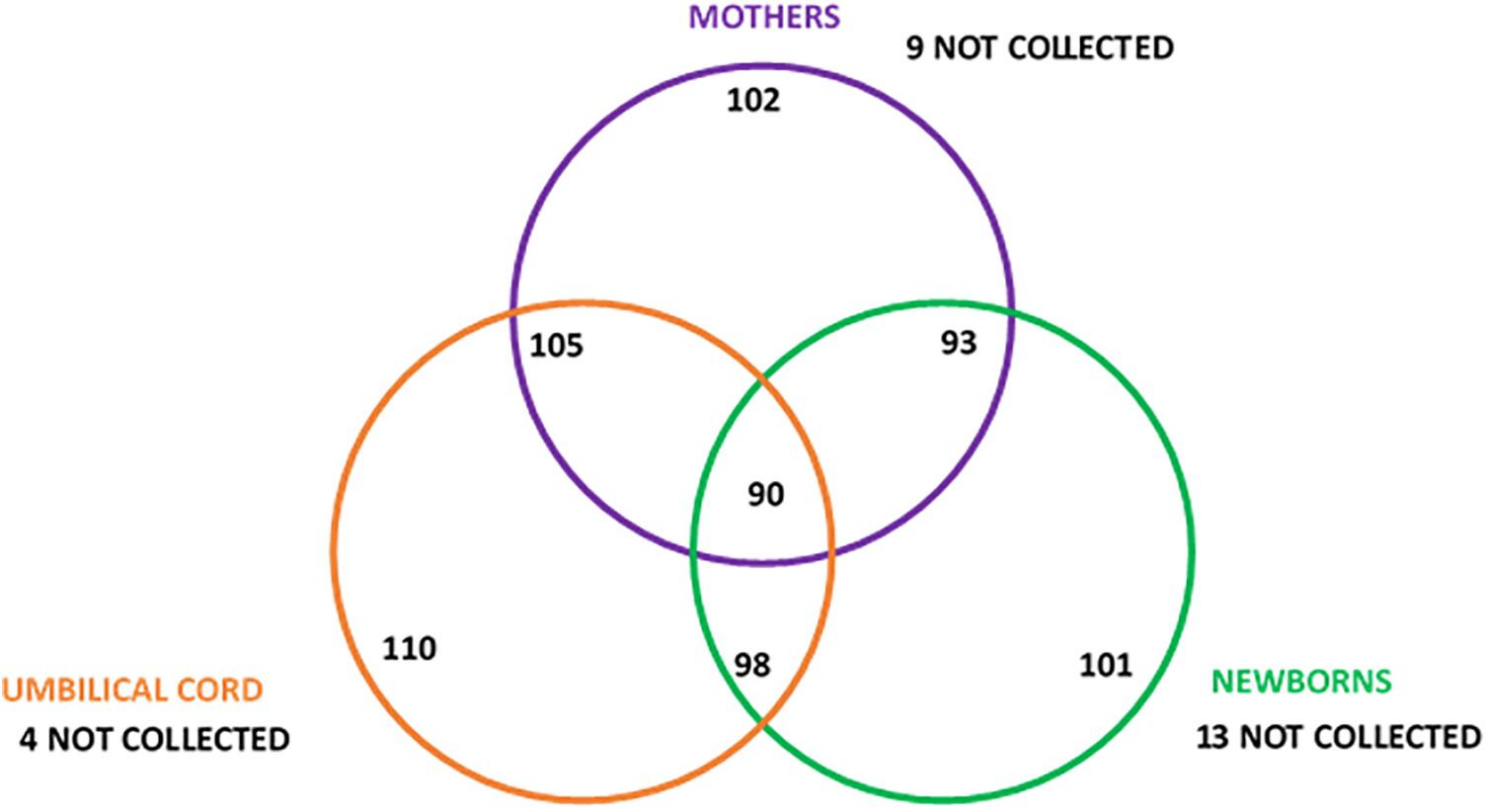
Venn diagram showing the total number of samples collected in each group. From the center outward, the 90 dyads with a complete sampling schedule (mother, child, and newborn) and the number of uncollected samples for each group are indicated.

Over a 15-month period, a total of 117 participating mothers with informed consent were registered, of which three were discarded because they decided not to continue in the study. Of the 114, only 102 samples were collected from mothers, 110 from UC and 101 from NB. It is worth mentioning that three mothers had twin births and in two of the three cases all samples were collected. Nineteen dyads had a complete sampling scheme (Mother, UC and NB) and the following combinations were obtained: mother + NB, mother + UC and UC + NB (Figure 1).

### 3.1 Demographic and gynecological data of the mothers

Demographic and gynecological data obtained from the medical histories of the 102 women whose samples were collected are described in Table 1. Overall, the age range was of 18–44 years, with more than 50% of them reporting a consensual union as marital status. Hospital O'Horan serves a population with a socioeconomic level middle-low without any type of other health security. Thus, completed education (elementary, or middle, or high school) was reported in percentages above 20%.

**Table 1:** Demographic and gynecological data of mothers categorized according to the type of immunity at the time of delivery or cesarean section.

Maternal demographics	No vaccination data *N* = 3 (3%)	Natural immunity *N* = 10 (9.8%)	Vaccination-based immunity *N* = 21 (20.5%)	Hybrid immunity *N* = 68 (66.5%)	*P* value
Average age (age range)	22.3 (19–26)	24 (19–32)	29 (19–43)	27 (18–44)	NA
Marital status					
Single	1	0	1	8	NA
Married	0	3	9	20	NA
Consensual Union	0	7	11	39	NA
No data	3	0	0	0	NA
Education					
No education	0	0	1	0	NA
Elementary	0	4	8	12	NA
Middle	1	2	5	28	NA
High	0	4	7	20	NA
University/college	0	0	0	7	NA
No data	2	0	0	1	NA
Ocupation					
Economically active women	0	1	3	6	NA
Homemaker	2	9	18	61	nNA
Student	0	0	0	1	NA
No data	1	0	0	0	NA
Obstetric data					
Primigravida	0	5	9	28	0.869*
Multigravida	2	5	12	40	
No data	1	0	0	0	–
Gestational evolution					
Normal (physiological)	1	8	17	61	NA
With complications (pathological)	0	1	2	6	NA
No data	2	1	2	1	-
Delivery type					
Eutocic^a^	0	4	1	8	0.021**
Dystocic^b^	2	6	20	60	
No data	1	0	0	0	–

Abbreviations: NA, not applicable.

a value obtained by Chi-square.

b Eutocic delivery: This is a delivery that occurs naturally, without complications, and without medical intervention.

c Dystocic delivery: This is a delivery with difficulties that requires medical intervention, such as the use of forceps or vacuum extraction, or even a cesarean section.

* Statistically significant (*P* < 0.05).

Obstetric data indicated that most of mothers were in their first pregnancy (41%), followed by mothers in their second or even fifth pregnancy (58%). Regarding pregnancy health, 85.2% of mothers had a normal pregnancy, while 8.8% experienced some complication. Despite the high percentage of normal pregnancies, cesarean delivery was reported in 86.2% of cases, with only 12.7% having spontaneous vaginal delivery. The main reasons for cesarean sections included preeclampsia (16%), fetal distress (11.4%), and other causes such as low fetal reserve, eclampsia, and placental abruption (each accounting for 4.5%). Notably, nearly half (49%) of cesarean sections were attributed to causes not specified (Supplementary Table S2).

Among the obstetric data, only the delivery type (eutocic or dystocic) showed a statistically significant relationship (*p* = 0.021) with respect to the immune status, indicating a possible correlation. Analysis of the variables included within the classification of pregnancy evolution (physiological or pathological) was not possible due to the low number of data points.

### 3.2 Neonatal clinical data

Data was obtained from the clinical history of the 101 newborns with a serum sample (Table 2).

**Table 2:** Data collected from the newborn's medical history categorized according to maternal immunity at the time of delivery or cesarean section.

	Uncollected maternal serum	No vaccine data	Natural immunity	Vaccine-induced immunity	Hybrid immunity	*P* value
	*n* = 7 (7%)	*n* = 3 (3%)	*n* = 10 (10%)	*n* = 22 (21.7%)	*n* = 59 (58.3%)	
Sex of newborn						
Male	3	0	7	10	36	NA
Female	3	2	3	11	22	NA
No data	1	1	0	1	1	NA
Gestational age						
Premature <37 GW	4	0	3	12	16	0.073*
Normal 37 to 42 GW	3	2	7	10	42	
No data	0	1	0	0	1	NA
Weight for gestational age						
Small	2	0	4	3	14	0.208*
Adequate	4	2	5	18	38	
Large	0	0	1	0	2	NA
No data	1	1	0	1	5	NA
APGAR^a^ (at one minute after birth)						
<7 Medical attention required	2	0	1	2	4	NA
≥7 Good health	4	2	9	19	52	NA
No data	1	1	0	1	3	NA
APGAR^a^ (at 5 minutes after birth)						
<7 Medical attention required	0	0	0	1	0	NA
≥7 Good health	6	2	10	20	57	NA
No data	1	1	0	1	2	NA
Received breast feeding						
Yes	3	1	4	14	42	0.204*
No	3	0	5	7	15	
No data	1	2	1	1	2	NA

NB, Newborn; GW, gestational Weeks, NA, not applicable.

* Chi-square.

a Test that evaluates the appearance, pulse, irritability, muscle tone, and respiration in the newborn.

A total of 55.4% were male and 40.6% were female. Regarding gestational age at delivery, although the majority of births (63.5%) occurred within a normal period of 37–42 weeks' gestation (WG), 34.5% were premature births within 37 WG.

The weight in grams of the newborn at birth was categorized according to the International Standards for Weight, Length and Head Circumference INTERGROWTH-21st project (97), which classifies newborns weights into: small (below the 10th percentile), adequate (between the 10th and 90th percentile) and large (above the 90th percentile) (Supplementary Table S1). The Pearson correlation analysis between gestational age and weight classification showed a strong positive correlation (*r* = 0.7159), with a *p* value < 0.0001 and alpha of 0.005.

At one minute after birth, 85% of newborns had an APGAR score of ≥7, while 9% scored <7. These results improved at five minutes, with 94% achieving a score of ≥7. Only one newborn did not show improvement at five minutes and required medical attention in the intensive care unit.

### 3.3 Detection of IgG and IgM antibodies against SARS-CoV-2

A total of 203 samples (from both mothers and newborns) were analyzed for the detection of IgM and IgG antibodies against SARS-CoV-2 by rapid test (QINGDAO HIGHTOP BIOTECH CO. LTD). The mothers' group had a 95% positivity rate (97 out of 102), and 5% were negative (5 out of 102). For the newborn group, 87.2% had a positive result (88 out of 101), while 12.8% were negative (13 out of 102). Only 1.96% of mothers tested positive for IgM antibodies against SARS-CoV-2 (2 out of 102).

### 3.4 Positivity to anti-N and anti-RBD IgG antibodies

**Figure 2:**
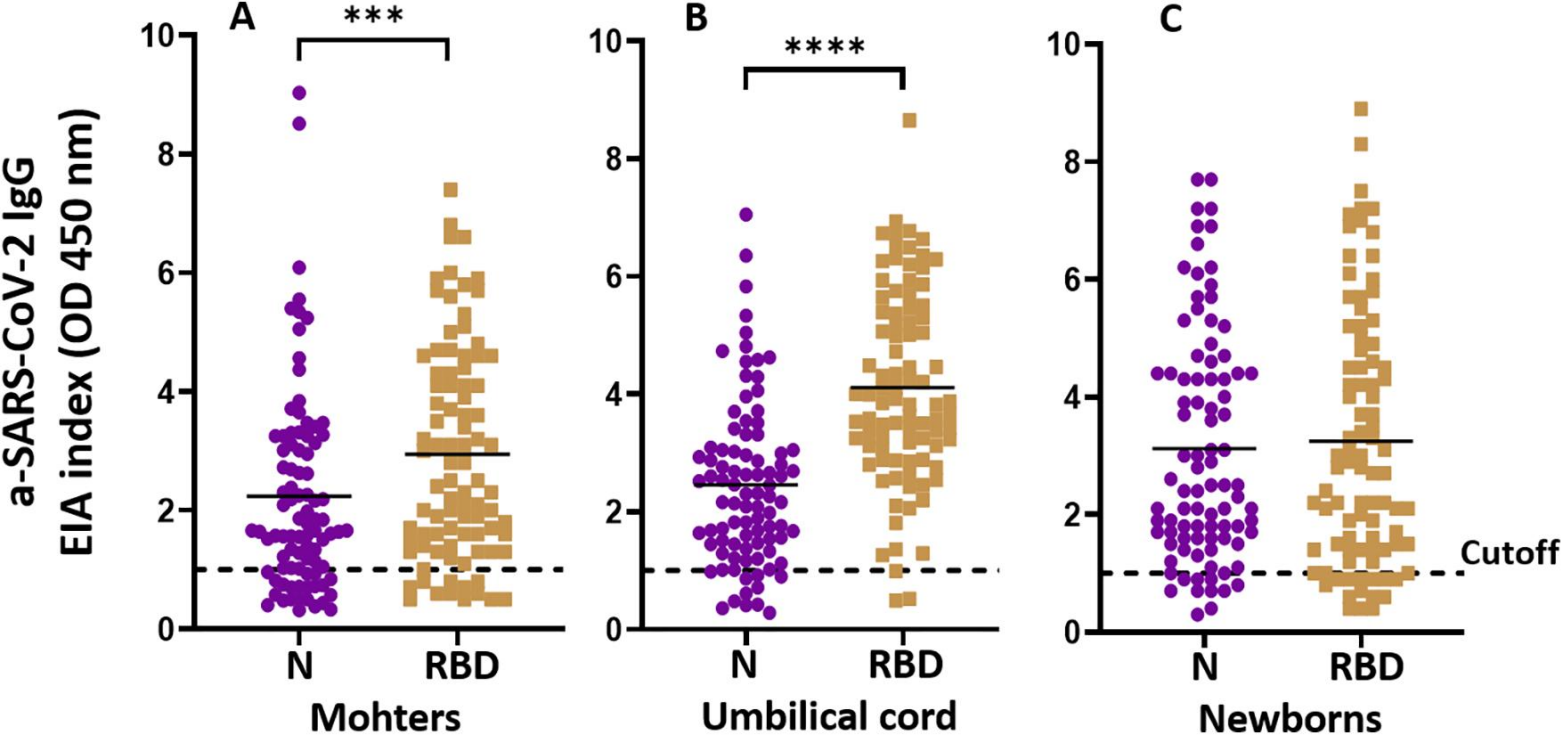
Comparison of EIA Index IgG anti-N vs. EIA Index IgG Anti-RBD in samples from **(A)** mother, **(B)** UC and **(C)** NB. (***) Indicates the significance value of *P* < 0.0002, (****) Indicates the significance value of *P* < 0.0001. Wilcoxon Comparison of Means Test. Prepared in Graph Pad Prism 8.

Anti-N and Anti-RBD IgG antibodies were determined in a total of 270 samples or 90 pairs (mother + UC + NB). Overall, the percentage of positivity for Anti-N IgG was lower in maternal samples than that observed for UC and NB. For Anti-RBD IgG, the percentage of positivity was highest for UC, followed by mothers and finally NB (Table 3). Seropositivity for Anti-N IgG was detected in 71.2% of mothers and 83.4% in NBs, and for Anti-RBD IgG, it was 85.5% in mothers and 81.2% in NB. Figure 2 describes Geometric Means (GM) of Anti-N and Anti-RBD IgG levels in samples from mother, UC, and NB.

**Table 3:** Percentage of seropositivity an geometric mean of nucleoprotein and RBD by ELISA.

Result	Mothers *n* = 90	GM	Umbilical cord *n* = 90	GM	Newborn *n* = 90	GM
IgG Anti-N n (%)						
Positive	64 (71.2)	2.232	76 (84.5)	2.455	75 (83.4)	3.121
Negative	26 (28.8)		14 (15.5)		15 (16.6)	
IgG Anti-RBD n (%)						
Positive	77 (85.5)	2.944	87 (96.6)	4.109	73 (81.2)	3.246
Negative	13 (14.5)		3 (3.4)		17 (18.8)	

N, nucleoprotein; RBD, receptor-binding domain: GM, Geometric mean.

Wilcoxon test was run to determine significant differences between GM of Anti-N and Anti-RBD IgG levels in each of the three groups. It was found that in both maternal and UC samples, there was a significant difference between GM of Anti-N and Anti-RBD IgG, with a significance of *P* = 0.0002 and *P* < 0.0001, respectively, indicating lower levels of Anti-N vs. Anti-RBD IgG; for the newborn group, no significant difference was found between the levels of these two IgGs. However, when GM of Anti-N IgGs were compared between groups (Mothers, UC and NB), the ANOVA test showed a significant difference between mothers and NB (*P* = 0.0022) indicating a higher level in NB. In contrast, for IgG anti-RBD, significant difference was observed between mothers and UC, and between UC and NB with significance values of *P* < 0.0001 and *P* = 0.0019 respectively, indicating a higher level in UC with respect to the levels in mothers and NB.

### 3.5 Immunization status of the mother-child pair: natural, vaccinated, or hybrid

Considering the ELISA seropositivity in the mothers, three immunization categories were established: natural immunity (NI), vaccination-induced immunity (IIV), and hybrid immunity (IH). Vaccination data were not recorded for three mothers (3.5%, 3/90) and therefore excluded from the analysis. As expected, the highest percentage of mothers (66.5%) showed hybrid immunity, followed by 20.5% with immunity from vaccination only and only 3% with natural immunity (Table 1). Similarly, the largest percentage of newborns (58.3%) presented hybrid immunity, followed by 21.7% with vaccine immunity and only 10% with natural immunity, in accordance with a similar pattern in mothers at the time of delivery (Table 2).

**Figure 3:**
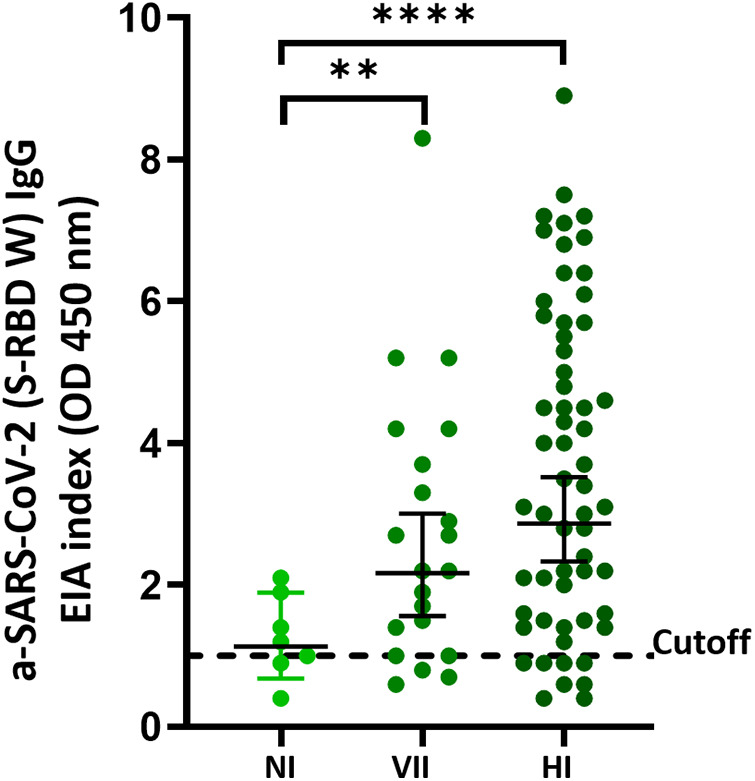
Geometric mean comparison of anti-RBD Index IgG EIA levels in newborns from mothers with different immunization statuses. NI, Natural Immunity; VII, vaccination-induced immunity; HI, Hybrid Immunization, (**) Indicates a significance level of *P* < 0.0002, (****) Indicates a significance level of *P* < 0.0001. Welch's ANOVA means comparison test. Prepared in Graph Pad Prism 8.

Statistical analysis was performed comparing anti-RBD IgG levels, given that these antibodies can be generated by both natural immunity and vaccination. ANOVA test using the Welch method was performed, assuming sample homogeneity and considering the different *n* sizes for each group (mother, UC, NB) according to their immunization status (Table 4). The Welch statistic helped determine the existence of significant differences between the geometric means of the Anti-RBD IgG EIA Index levels with multiple comparisons among the t mother, UC an NB groups. However, the result indicated significant differences between the immunization status only for the NB group with a statistic value of W = 21.82, and a *P* value <0.0001. Then, a Holm-Sidak's multiple comparison test was run only for NB data and the results showed significant differences between natural immunity vs. vaccination immunity (*t* = 3.09 and *p* = 0.0093), and between natural immunity vs. hybrid immunity (*t* = 6.54 and *p* < 0.0001) (Figure 3). GM for vaccination immunity and hybrid immunity were significantly higher (mean difference = −1.462 and −2.4, with an alpha value of 0.05).

**Table 4:** Immunization status of mothers and ANOVA test for EIA Index mean in newborn.

Recorded cases	No vaccination data	Natural Immunity	Vaccination-based Immunity	Hybrid Immunity
90 (100%)	3 (3.5)	7 (7.7)	21 (23.3)	59 (65.5)

### 3.6 Anti-RBD IgG levels in vaccinated mothers

**Figure 4:**
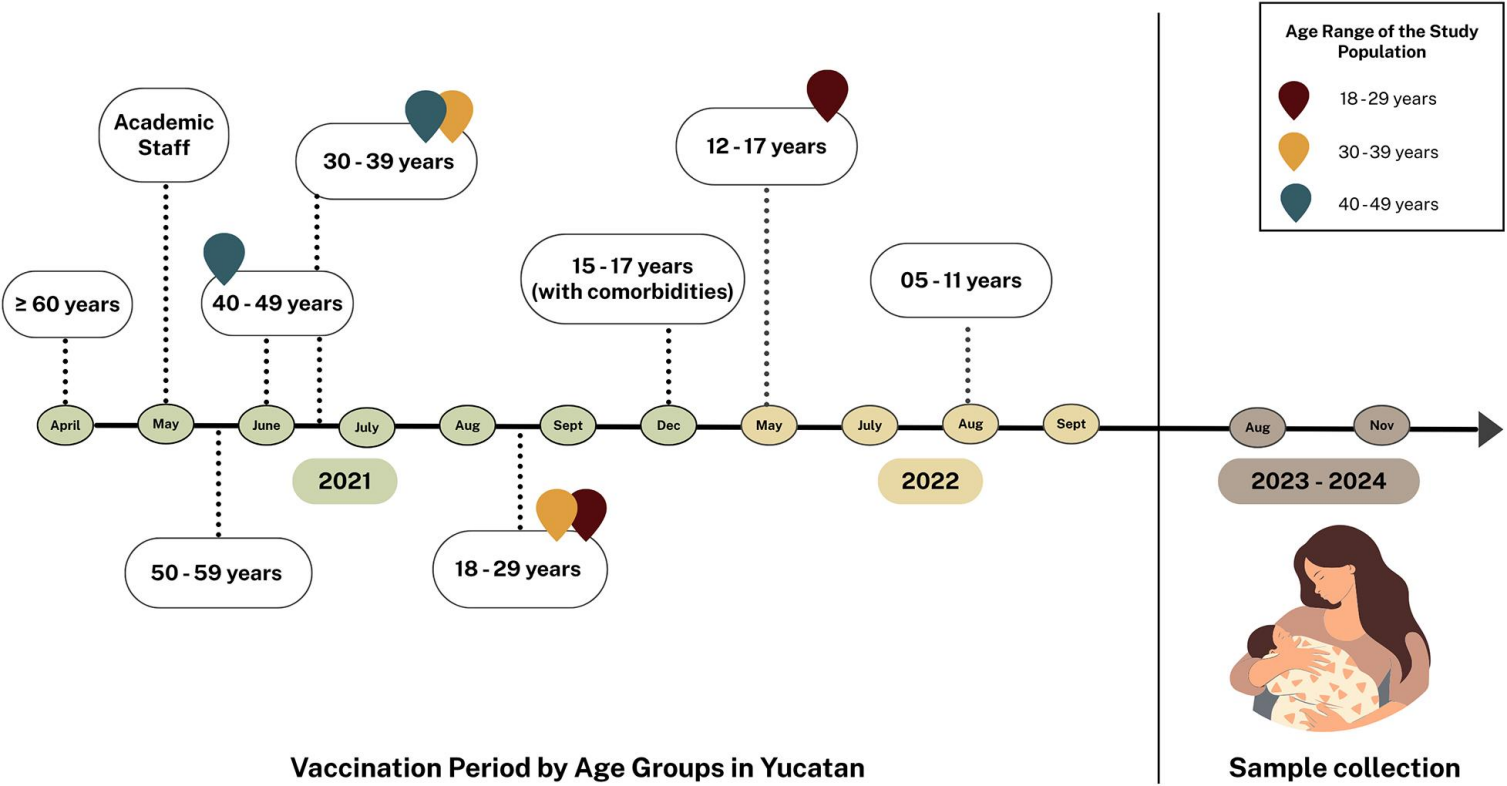
Timing and age of vaccination of recruited mothers during COVID-19 vaccination campaigns in yucatan. The vaccination periods by age group in 2021 and 2022, as specified by public health vaccination campaigns are shown (top panel), and the ages of the mothers during vaccination prior to being included in the study are specified in the bottom panel.

A variable of interest to consider for these pairs was the receipt of any SARS-CoV-2 vaccine, regardless of the biological product administered. Table 5 summarizes the overall picture of the vaccines administered to the mothers. A total of 89% reported having received at least one dose of the SARS-CoV-2 vaccine, while 8% reported none, and only 3% had no vaccination information. Among the mothers who reported receiving a vaccine, 15% indicated receiving one dose, followed by 38.8% with two doses, and finally 43.8% with three or more doses. The most frequently administered was AztraZeneca AZD1222 (VaxZevria). It is relevant to mention that SARS-CoV-2 vaccination in the women included in this study was not performed during pregnancy; vaccination occurred prior to pregnancy in accordance with the established age groups during the vaccination campaign in the state of Yucatán from April 2021 to December 2022. Figure 4 shows the age of mothers during vaccination campaigns in Yucatan.

**Table 5:** Vaccination data reported by mothers.

Vaccinated^a^	One Dose	Two Dose	≥Three Dose
*n* = 80 (89%)	*n* = 12 (15%)	*n* = 31 (38.8%)	*n* = 35 (43.8%)
Astrazeneca vaxzevria (AZD1222)	10 (83.4%)	19 (61.3%)	22 (70%)
Cansino convidecia (AD5-NCOV)	1 (8.3%)	3 (9.7%)	0
Pfizer biontech (BNT162B2)	1 (8.3%)	6 (19.3%)	2 (6.8%)
Sinovac biotech (CORONAVAC)	0	1 (3.2%)	3 (10%)
Moderna spikevax (ANDUSOMERáN)	0	1 (3.2%)	0
AZD1222 + AD5-NCOV	0	1 (3.2%)	3 (6.6%)
AZD1222 + BNT162B2	0	0	4 (3.3%)
AZD1222 + CORONAVAC	0	0	1 (3.3%)
Not vaccinated *N* = 7 (8%)			

a Two mothers, (2.5%) reported being vaccinated but did not provide information on the dose or type of vaccine.

Three mothers did not provide information on the SARS-COV-2 vaccine.

**Figure 5:**
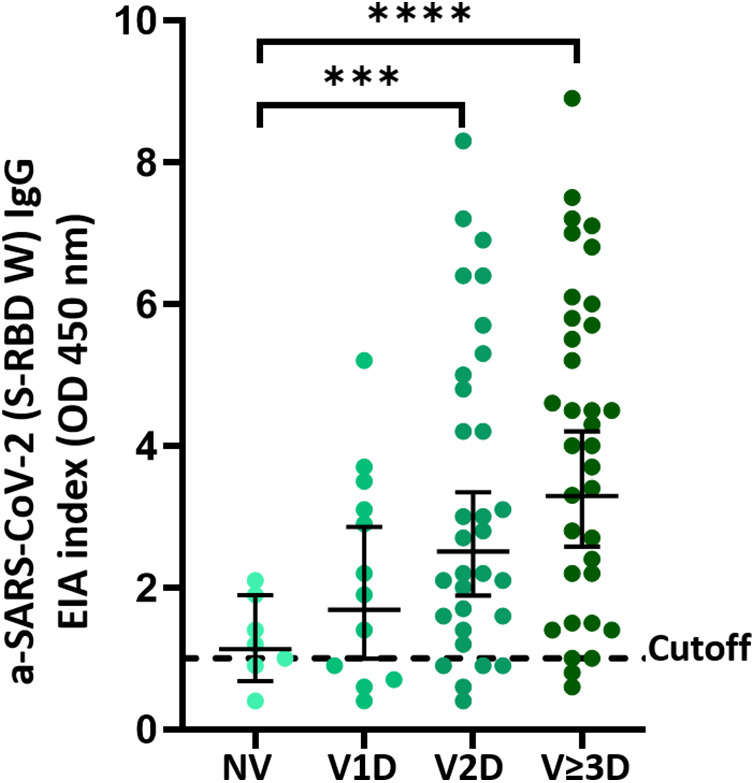
Comparison of the geometric means of the anti-S-RBD Index IgG EIA levels of newborn sera from mothers vaccinated with 1, 2, and ≥3 doses compared to the control newborns of unvaccinated mothers. NV, not vaccinated; V1D, vaccinated 1 dose; V2D, vaccinated 2 doses; V ≥ 3D, vaccinated ≥3 doses, (***) Indicates a significance level of *P* = 0.0003, (****) Indicates a significance level of *P* < 0.0001 using Dunnet's test. Prepared in Graph Pad Prism 8.

Based on the number of vaccines doses in mothers, an ANOVA test using the Welch method was performed to determine significant differences between Anti-S-RBD IgG Index EIA levels for NB, comparing vaccine doses against the control (non-vaccinated). The test showed significant difference (W = 15.32, *P* < 0.0001). The Dunnett's correction also showed significance when mothers received two doses of vaccine (*t* = 4.35, df = 36, *p* = 0.0003) as well as≥3 doses compared to the control group (*t* = 6.27, df = 40, *p* < 0.0001) (Table 6 and Figure 5). The means of the groups with two and ≥three vaccine doses were significantly higher than the mean of the control group [mean difference = 1.98 and 2.76, 95% CI = [−3.057 to −0.9037] and [−3.799 to −1.721] respectively].

**Table 6:** Comparison of the mean anti-SARS-CoV-2 IgG index by EIA in sera of newborns categorized by number of vaccine doses in mothers with the unvaccinated control group. Dunnett statistical values.

**Dunnett test**	**Difference in means**	**Significance**	**Adjusted P**		
NV VS. V1D	−0.9369	No	0.1408		
NV VS. V2D	−1.980	Yes	0.0003		
NV VS. V ≥ 3D	−2.760	Yes	<0.0001		

**Test detail**	**Mean 1**	**Mean 2**	**Standard Error Difference**	**n1**	**n2**	**T**
NV VS. V1D	1.271	2.208	0.4864	7	12	1.926
NV VS. V2D	1.271	3.252	0.4543	7	31	4.359
NV VS. V ≥ 3D	1.271	4.031	0.4397	7	35	6.277

### 3.7 Neutralization assays

Neutralizing antibody titers were measured at the time of delivery in 135 samples, corresponding to 45 pairs (mother, umbilical cord, and newborn), representing 50% of the total pairs analyzed for Anti-N and Anti-RBD IgG antibodies. Among these 45 pairs, 4 were classified as natural immunity, 18 as vaccination-induced immunity, and 23 as hybrid immunity. The overall positivity rates for neutralization of the Wuhan variant pseudovirus were 75.6% in mothers, 98% in umbilical cord blood, and 62.3% in newborns.

**Figure 6:**
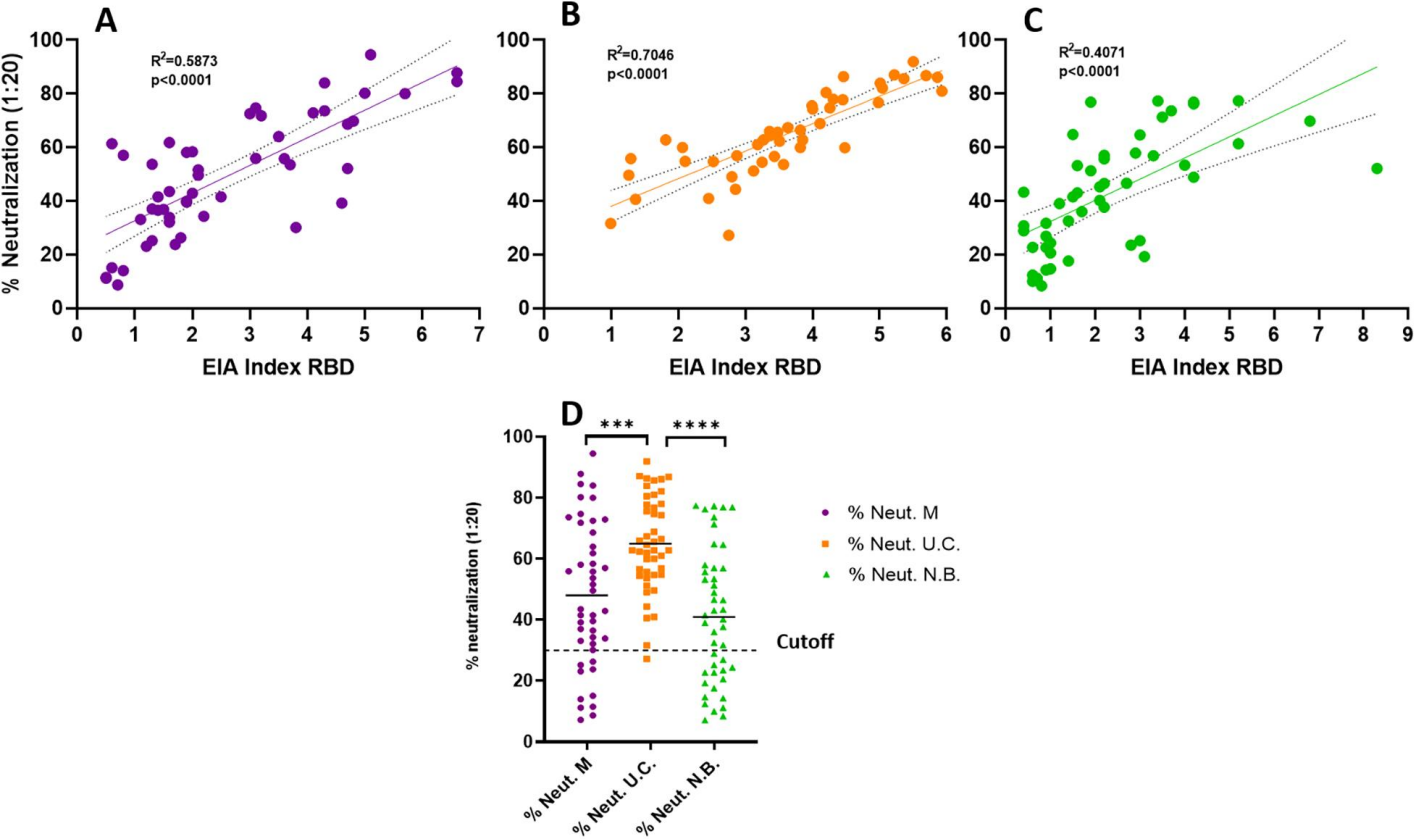
Correlation analysis between EIA Index RBD and % of neutralization for (5A) mothers, (5B) UC and (5C) NB. Percentage of GM between mothers, UC and NB (5D). (***) Indicates a significance level of *P* < 0.0005, (****) Indicates a significance level of *P* < 0.0001 Tukey's ANOVA means comparison test.

Using the IgG antibody titer data expressed as EIA Index and the neutralization percentages, a correlation analysis was performed using linear regression (Figures 6A–C). Analyses showed a positive correlation between the ELISA Index value of Antibody titers for RBD and the percentage of neutralization with *P* < 0.0001 values and positive *R*^2^ values of 0.58, 0.7 and 0.4 in mothers, umbilical cord and newborns respectively.

Comparison of the geometric means of the neutralization percentages in each of the three groups (mothers, UC, and NB) using ANOVA (Figure 6B) revealed a significant difference between the UC value and those of mothers and NB. The geometric mean of UC (64.94) was higher than that of mothers (48.05) and NB (40.92), with significance levels of *P* = 0.0005 and *P* < 0.0001, respectively.

In the analysis comparing the geometric means of neutralization percentages according to maternal immunity status at the time of delivery or cesarean showed no significant difference in any of the three groups (Supplementary Figure S1). Equally, the analysis performed to determine if there is a significant difference between the geometric means of neutralization percentages according to the number of vaccine doses received by the mothers indicated no significant difference (Supplementary Figure S2).

## 4 Discussion

This study reports the presence of IgG antibodies against SARS-CoV-2 in pregnant women and paired samples from newborns and umbilical cord at the time of delivery or cesarean section. Our findings indicate that regardless of the timing of maternal vaccination and the time elapsed since their last natural infection with SARS-CoV-2, 95% of pregnant women still present IgG antibodies against this virus. These antibodies were transferred to the newborn through the placenta in 87% of cases.

During the antibody response to SARS-CoV-2 infection, IgM antibodies peak after the week of infection and can be maintained for up to a month until a gradual decrease (12). The enrolled mothers in this study did not indicate a history of natural infection during pregnancy, or any risk condition associated with infection with the SARS-CoV-2 virus as has been reported (5). Two mothers showed positivity for IgM antibodies by rapid test, suggesting a recent asymptomatic infection. In contrast, samples from UC and NB were negative for IgM antibodies, consistent with reported evidence that maternal IgM antibodies do not cross the transplacental barrier (12, 13).

During the COVID-19 pandemic, rapid antibody detection tests as evidence of effective vaccination or natural infection gained significant global relevance (14, 15). In this study, utilizing this strategy, a rapid antibody detection test was performed on samples from mothers and newborns. A 91% positivity rate for IgG antibodies was obtained from a total of 203 samples analyzed. Concordance was observed between the positivity results obtained by the rapid test and those obtained by ELISA; two of three samples that gave a negative result by rapid test coincided with a negative result by ELISA for both Anti-N and RBD, and only one gave a positive result for Anti-RBD by ELISA, reinforcing the greater sensitivity of ELISA tests (16, 17). The high percentage of seropositivity was expected within the population, due to the post-pandemic period at which samples were collected, with higher probability of exposure to natural infection, and also the good vaccination program within the general population (18).

Regarding IgG levels and maternal-fetal transfer during pregnancy, very little transfer has been reported around 12 weeks of gestation (WG) (19), with levels present in the villous stroma of the placenta corresponding to between 8 and 10 WG (20). Since most of the IgG in fetal blood is of maternal origin, its concentration reflects transport from the mother. This leads to fetal IgG levels remaining low until the second trimester (21, 22). Fetal IgG levels are known to increase from the second half of the second trimester (21, 22), between 22 and 26 weeks of pregnancy, with an average increase in fetal IgG concentration from 1.4 ± 0.7 g/L at 17-22 weeks of pregnancy (approximately 10% of maternal concentration) to 5.6 ± 1.1 g/L at 28–32 weeks of pregnancy (approximately 50% of maternal concentration). Fetal IgG levels are also known to continue to increase during the third trimester. At term, fetal IgG levels often slightly exceed maternal levels (23). Several factors influence the efficiency of IgG transfer during pregnancy, including concentration, IgG subclass, glycosylation, maternal infections, and antigen specificity. The integrity of the placenta also plays a vital role in mediating this transfer (24).

Many studies have shown that infants can effectively receive antibodies produced by the mother after SARS-CoV-2 infection during pregnancy (24–26). In a study of placental antibody transfer, it was observed that total IgG transfer levels in infected pregnant women were no different from those in uninfected pregnant women. The transmission of placental antibodies unrelated to SARS-CoV-2 is normal in the presence of maternal SARS-CoV-2 infection, but placental transfer of SARS-CoV-2-specific antibodies is reduced (27).

Furthermore, levels of Anti-N IgG antibodies were found to be lower than those of Anti-RBD, which is consistent with previous reports (28). The RBD region of the SARS-CoV-2 Spike protein is the primary site of interaction with the specific ACE2 receptor, and is therefore a very important and primary antigenic site on the virus. In contrast, nucleoprotein is a protein primarily associated with viral packaging, is highly conserved, and is less antigenically exposed (29, 30).

In this study, the mothers' immunity status (natural infection, vaccination, hybrid) was categorized at the time of delivery or cesarean section based on the results of the ELISA test. The detection of anti-N antibodies has been associated with natural infection prior to vaccination (31, 32). The low positivity for anti-N antibodies could be due in part to the high percentage of exposure the mothers had to both natural infection and vaccination up to the time of sampling. Likewise, previous studies have reported that anti-N antibodies decrease over time (33, 34). Immunity through vaccination alone was identified in 23% of the maternal population, and although it represents an almost four-fold increase compared to cases of natural immunity, a reduction in anti-RBD antibody levels has also been reported as part of the natural process of the immune response to SARS-CoV-2 (33). Finally, 65.5% (59/90) of mothers were classified as having hybrid immunity, results that reinforce the important role of the immune response due to natural infection, which is strengthened by vaccination (4, 35). Even more important is the role it plays in the transfer of antibodies to newborns during pregnancy. According to data from the Government of Mexico, by 2021, Yucatán reached 86% coverage in people over 18 years of age (18).

The Omicron subvariant XBB.1 was in circulation in Yucatan during the months of clinical sample collection. Studies have shown the durability of hybrid immunity after infection with the omicron variant (35, 36) which are consistent with the results of this study.

Another important aspect of this study is the significant difference observed in the levels of IgG antibodies detected in newborns when mothers received more than two vaccine doses. Considering that AstraZeneca vaccine was administered to 82.5% of vaccinated mothers, this suggests a robust and long-lasting IgG antibody response, consistent with previous studies (37, 38).

Finally, the study identified a positive correlation between IgG and neutralizing antibodies. The percentage of neutralization was higher in UC samples (98%) and higher in titer (% GM of 65%), similar to the anti-RBD IgG antibodies obtained by ELISA. These data are explained by the fact that blood from UC carries higher levels of hematocrit as part of the oxygen and nutrients requirement for the baby. Thus, blood from the UC is considered reflects fetal blood just before birth, with higher levels of components, including IgG antibodies, even higher than in maternal blood or in the NB blood few hours after birth (39–41).

It is interesting to mention, that in contrast to the results of total IgG antibodies against RBD, no significant difference in neutralization was observed, between mothers, UC and NB when compared by type of immunity (natural vs. vaccination vs. hybrid), nor when mother, UC and NB were compared according to the number of vaccine doses received by the mother. Waning immunity has been extensively reported for SARS-CoV-2 after vaccination and with the occurrence of new variants of concern (42–45). Previous studies have shown that functional antibodies are not always present at the levels expected based on antibody titers, indicating that while generally well-correlated with maternal serum antibodies at the time of delivery, the relative activity of antibodies in cord blood are distinct (46).

The overall positivity rates for neutralization of the Wuhan variant pseudovirus were lower in mothers and newborns. The mRNA vaccines targeting the ancestral S protein elicited heavily cross-reactive antibody response with no detectable production of antibodies that target novel epitopes on the RBD recent variants as BA.5 or XBB (47). This could represent, in our study, a possible explanation for the difference found between the geometric means of the neutralization, albeit the circulating variant in Yucatan from January to September 2023 was XBB.1, coinciding with the start of the collection of the pairs in our study.

In the analysis of antibody levels between preterm and term newborns, no significant difference was found between the geometric means, nor was there a correlation between prematurity and low antibody levels or low neutralization percentages. It is relevant to note that, in this study, the group of newborns classified as preterm were born around weeks 35 and 36 of gestation, relatively close to week 37, which is considered the cutoff point for term newborns. This could explain the lack of a marked difference in antibody levels and/or neutralization percentages.

A limitation of this study was that, unfortunately, only pseudoviruses of the Wuhan variant were available, so neutralization assays could not be performed against other more recent variants, such as Omicron subvariant XBB.1, or any other subvariant circulating closer to the period of serum collection. However, knowing that all vaccines were focused on the S protein of the Wuhan variant, we considered that for this initial experimental approach, the challenge with only these pseudoviruses would be sufficient.

Finally, an important aspect to consider is the determination of IgG isotypes for a better understanding of the immune system's response. Kizel P et al. in 2026, determined that the vaccine response preferentially showed the presence of the IgG1 and IgG3 subclasses, and that patients with persistent or chronic infection presented elevated levels of IgG4 and IgE (48) This could explain the observed IgG antibody levels and neutralization percentages in our group; however, the lack of isotype determination was another limitation of this study, which is of interest for future research.

## Data Availability

The raw data supporting the conclusions of this article will be made available by the authors, without undue reservation.
