# Supplementary material for: Maternal immunization status and SARS-CoV-2 antibody transfer to neonates at birth
Source: Front Pediatr. 2026 Jul 1;14:1824042. doi: 10.3389/fped.2026.1824042 (PMC13368714; doi:10.3389/fped.2026.1824042)
Supplement: Supplementary file 1 [file Supplementaryfile1.docx]

Supplementary

Table S1. Weight categorization according to gestational age and sex of the newborn.

| **Gestational age*** | **Sex** | **Small**  **n (weight in grams)** | **Adequate**  **n (weight in grams)** | **Large**  **n (weight in grams)** |
| --- | --- | --- | --- | --- |
| **Premature** <37 WGA  **25 (24.8%)** | Male | 2 (1,130 – 1,780) | 7 (1,570 – 2,460) | 1 (3,480) |
|  | Female | 8 (850 – 2,330) | 7 (1,490 – 2,910) | 0 |
|  | | | | |
| **Normal** 37 a 42 WGA  **64 (63.4%)** | Male | 7 (2,070 – 2,990) | 33 (2,150 – 3,970) | 1 (4,080) |
|  | Female | 7 (1,730 – 3,100) | 15 (2,260 – 3,890) | 1(3,900) |
| * Gestational age data were not available for 12 (11.8%) newborns. WGA, Weeks of Gestational Age. | | | | |

Table S2. Reasons for performing a cesarean section, categorized according to the type of immunity at the time of delivery or cesarean section.

| REASON FOR CESAREAN ^a^ | No vaccination data n=3  (3%) | Natural Immunity n=10  (9.8%) | Vaccination-based Immunity n=21  (20.5%) | Hybrid Immunity n=68  (66.5%) |
| --- | --- | --- | --- | --- |
|  |  |  |  |  |
| PCD | 1 | 0 | 1 | 2 |
| Fetal distress | 0 | 0 | 5 | 5 |
| Low fetal reserve | 0 | 0 | 1 | 3 |
| Preeclampsia | 1 | 3 | 0 | 10 |
| Eclampsia | 0 | 1 | 2 | 1 |
| Rupture of membranas | 0 | 0 | 0 | 1 |
| Placental abruption | 0 | 0 | 2 | 2 |
| Iterative ^b^ | 0 | 0 | 0 | 1 |
| Other cause | 0 | 2 | 9 | 32 |
| No data | 0 | 0 | 0 | 3 |
| Abbreviations: PCD, Cephalopelvic Disproportion  ^a^ Percentages are based on the number of reported cesarean sections  ^b^ Cesarean performed in a woman with a previous cesarean section in a previous pregnancy. | | | | |


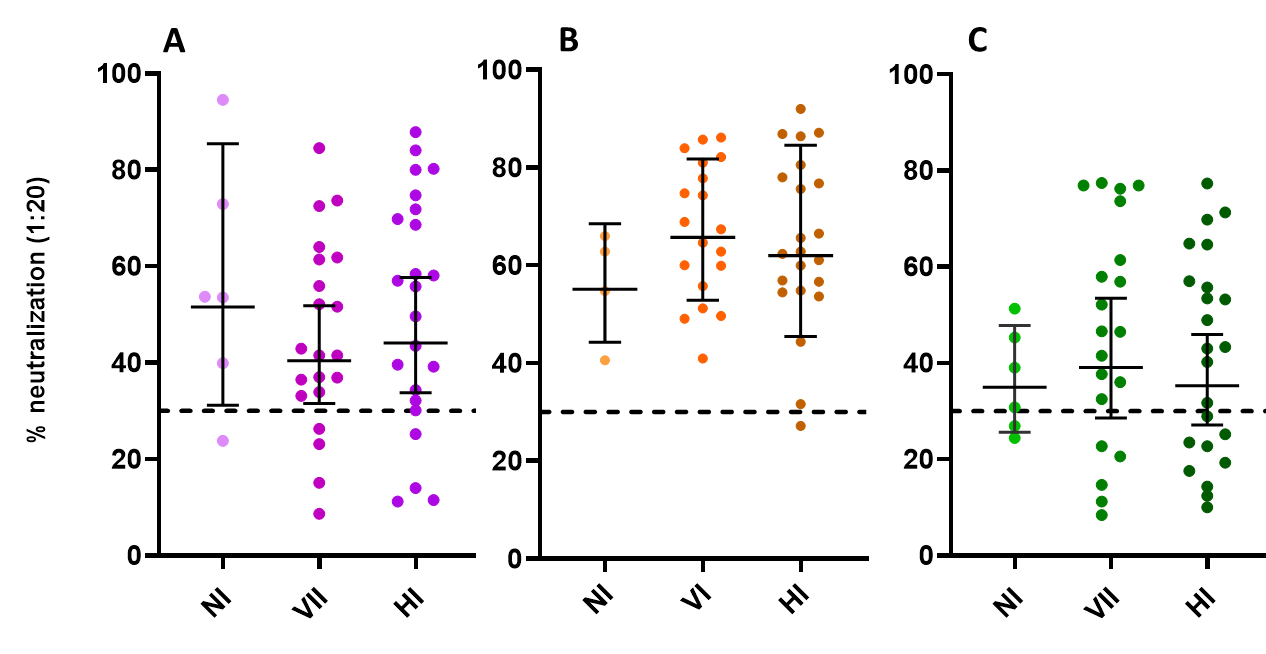


Figure S1. Comparison of geometric mean of neutralization percentages in samples of A) Mothers, B) Uterine Units, and C) Newborns according to the type of maternal immunity at the time of delivery or cesarean section. NI: natural immunity, VII: vaccination-induced immunity, or HI: hybrid immunity. ANOVA test for multiple comparisons of means using Dunnett's method. Prepared in GraphPad Prism 8.


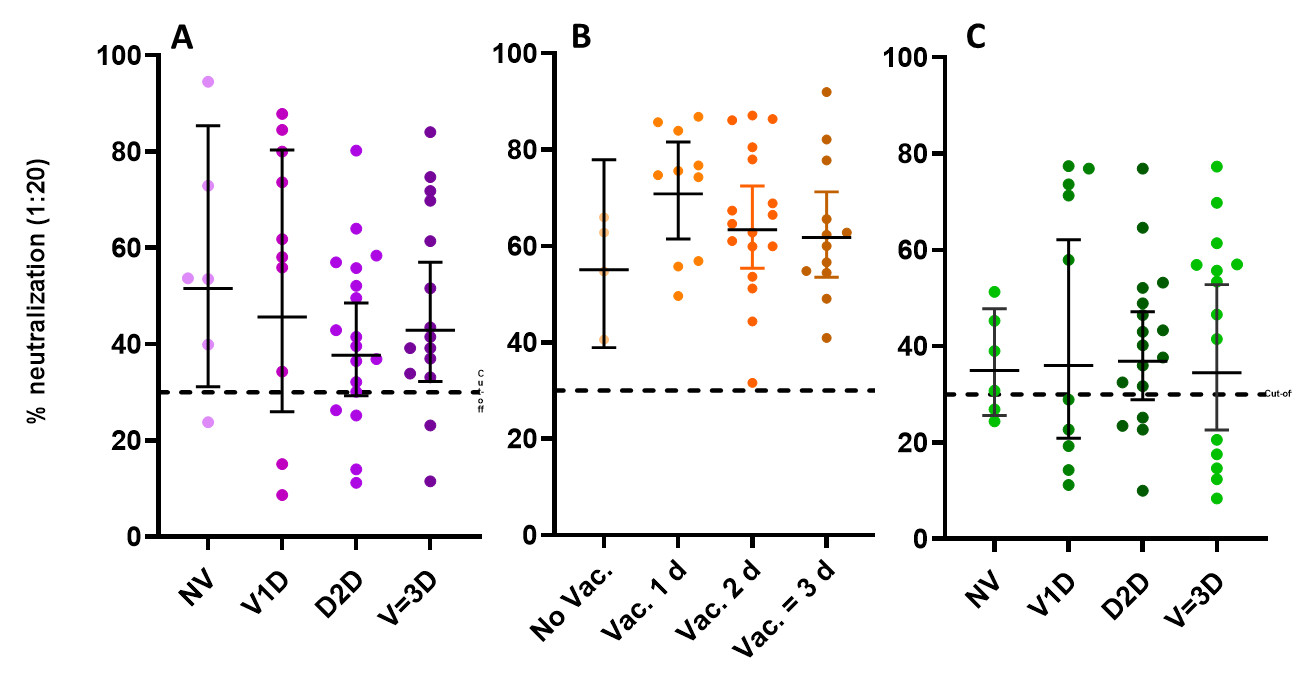


Figure S2. Comparison of geometric mean of neutralization percentages in samples of A) Mothers, B) C.U. and C) RN according to the number of doses received by the mother. ANOVA test for multiple comparisons of means by Dunnett's method. Prepared in GraphPad Prism 8.
